# Glyco-conjugated bile acids drive the initial metaplastic gland formation from multi-layered glands through crypt-fission in a murine model

**DOI:** 10.1371/journal.pone.0220050

**Published:** 2019-07-26

**Authors:** Danielle Straub, Ronald P. J. Oude Elferink, Peter L. M. Jansen, Jacques J. G. H. M. Bergman, Kaushal Parikh, Kausilia K. Krishnadath

**Affiliations:** 1 Center for Experimental and Molecular Medicine (CEMM), Academic Medical Center, Amsterdam, The Netherlands; 2 Tytgat Institute for Liver and Intestinal Research, Academic Medical Center, Amsterdam, The Netherlands; 3 Department of Gastrointestinal and Liver Disease, Academic Medical Center, Amsterdam, The Netherlands; 4 Department of Gastroenterology, Academic Medical Center, Amsterdam, The Netherlands; 5 MRC Human Immunology Unit, Weatherall Institute of Molecular Medicine, University of Oxford, John Radcliffe Hospital, Oxford, United Kingdom; National Cancer Institute, UNITED STATES

## Abstract

Bile acid reflux is known to be associated with the development of Barrett’s esophagus and esophageal adenocarcinoma (EAC), yet the role of specific bile acids and the mechanism behind the metaplastic changes is unclear. Here, we demonstrate that multi-layered glandular structures at the squamo-columnar junction in mice contain multiple cell lineages, which resemble the human esophageal submucosal gland ducts. Exposing mice to patient’s refluxates induced expansion of multi-layered glandular structures and development of columnar metaplasia at the squamo-columnar junction. The glycine conjugated bile acids induced an intestinal type of metaplasia more typical for Barrett’s esophagus. Through lineage tracing, we excluded the involvement of K5^+^, DCLK1^+^, and LGR5^+^ progenitor cells as the primary source in the development of the glandular metaplastic epithelium. We show that the mechanism behind development of metaplasia involves crypt fission and may be independent of stem cell proliferation. Our findings support the hypothesis that in humans, BE arises from non-squamous cells residing in submucosal gland ducts and that induction of intestinal type of metaplasia is most effectively induced by glycine-conjugated bile acids. These novel insights may lead to more effective strategies to prevent development of Barrett’s esophagus and esophageal adenocarcinoma.

## Introduction

The incidence of esophageal adenocarcinoma (EAC) is one of the fastest rising in Western countries “[1]”. Duodeno-gastric-esophageal reflux disease (DGERD) and Barrett’s esophagus (BE) are predisposing conditions for EAC “[2–4]”. It is thought that DGERD causes damage and inflammation of the normal squamous epithelium and enhances development of columnar metaplasia “[5]”. This epithelial metaplasia, or BE, carries an increased risk for EAC. Proton pump inhibitors (PPIs) the mainstay of treatment, helps to relieve reflux symptoms, but only partially reduce the risk of EAC in patients with BE “[6]”. Similar results are seen when PPI use is combined with aspirin chemoprevention therapy “[7]”. A search for alternative, more effective preventive therapies is warranted.

It has been shown that bile acids (BAs) play an important role in the DGERD-BE-dysplasia-EAC sequence “[2,8]”. Indeed, the concentration of BAs in refluxates is higher in BE patients compared to healthy subjects “[3,4]” and an increased severity of mucosal damage has been observed when BAs were found in combination with acid, compared to acidic reflux alone “[9]”. BAs are produced in the liver and are conjugated with either taurine or glycine before being secreted in the bile pool and released in the duodenum “[10]”. Although BAs are thought to be involved in the pathogenesis of BE, their specific role have been poorly studied.

Another critical, yet unsolved, issue in the development of BE metaplasia is that the initial process underlying metaplasia at the originating site is still unclear. This hampers development of adequate BE and EAC models and development of adequate therapies. Several hypotheses have been proposed. These include migration of columnar gastric (stem) cells “[11]”, trans-differentiation of esophageal squamous cells “[12]” or involvement of bone marrow cells “[13]”. It has been observed that BE and EAC can develop in (transgenic) mice such as the IL1beta “[14]” and the p63 knockout mice “[15]”. As mice are thought to lack submucosal glands “[16]”, the origin of these lesions has been attributed to embryonic remnants or to upward migration of stomach stem cells towards the squamo-columnar junction (SCJ) “[11,12]”. Interestingly, administration of bile acids such as deoxycholic acid enhanced the metaplasia-carcinogenesis process in IL1beta transgenic mice “[14]”. More recently, Jiang *et al* further explored the SCJ and found a transitional columnar type of epithelium, consisting of (p63^+^K5^+^) basal cells and (K7^+^) luminal cells “[17]”. They showed that intestinal metaplasia arose from the transitional multilayered epithelium at the SCJ in Krt5-CDX2 transgenic mice. These studies point toward stem cell niches at the SCJ in mice, yet the underlying mechanism and the exact pathophysiological stimuli driving the metaplastic process in patients with reflux are still unclear.

The aim of our study was to investigate the effects of bile acids from refluxates of Barrett patients on the development of columnar and intestinal type of metaplasia and understand the initial mechanism underlying this process. We first compared the structure of the multi-layered glandular structures (MLGS) at the squamo-columnar junction in mice to that of the human esophageal submucosal glands (SMG) and submucosal gland ducts (SMGD). We next characterized the BA composition in refluxates of BE patients by high performance liquid chromatography (HPLC). We tested the effects of human refluxates, and of individual taurine- and glycine-conjugated BAs in mice and observed the development of columnar epithelium from the MLGS. The expansion of MLGS was in accordance with the process of crypt fission. We found that glycine-conjugated bile acids were able to induce the intestinal type of columnar epithelium resembling human BE. Using lineage tracing we have not only excluded K5^+^ squamous progenitors as the source of the columnar metaplasia, but also identified several columnar progenitor cells in the MLGS that could potentially give rise to the metaplastic glandular epithelium at the SCJ.

## Results

### Mouse multi-layered glandular structures show homology to human esophageal submucosal gland duct and human multilayered epithelium

In a previous publication we showed that reflux of bile in a surgical mouse model resulted in the development of intestinal metaplasia at the esophago-jejunostomy “[18,19]”. In these mice we also observed development of multi-layered glandular structures (MLGS) and glandular epithelium at the squamo-columnar junction (SCJ) in the stomach (S1 Fig). Here, we first compared the structure of these MLGS and glandular epithelium to that of human esophageal submucosal gland ducts (SMGD) and early columnar metaplastic lesions “[20]”.

In the human esophagus, submucosal glands (SMG) function as a defence mechanism. Serous-producing acini are connected to mucus-producing tubules, which merge into collecting ducts to excrete their mucus into the lumen “[21]”. The human SMGD is lined in the proximal two-thirds by flattened cuboidal cells, which undergoes a gradual transition, from two layers to a stratified squamous epithelium towards the luminal surface “[22]”.

**Fig 1:**
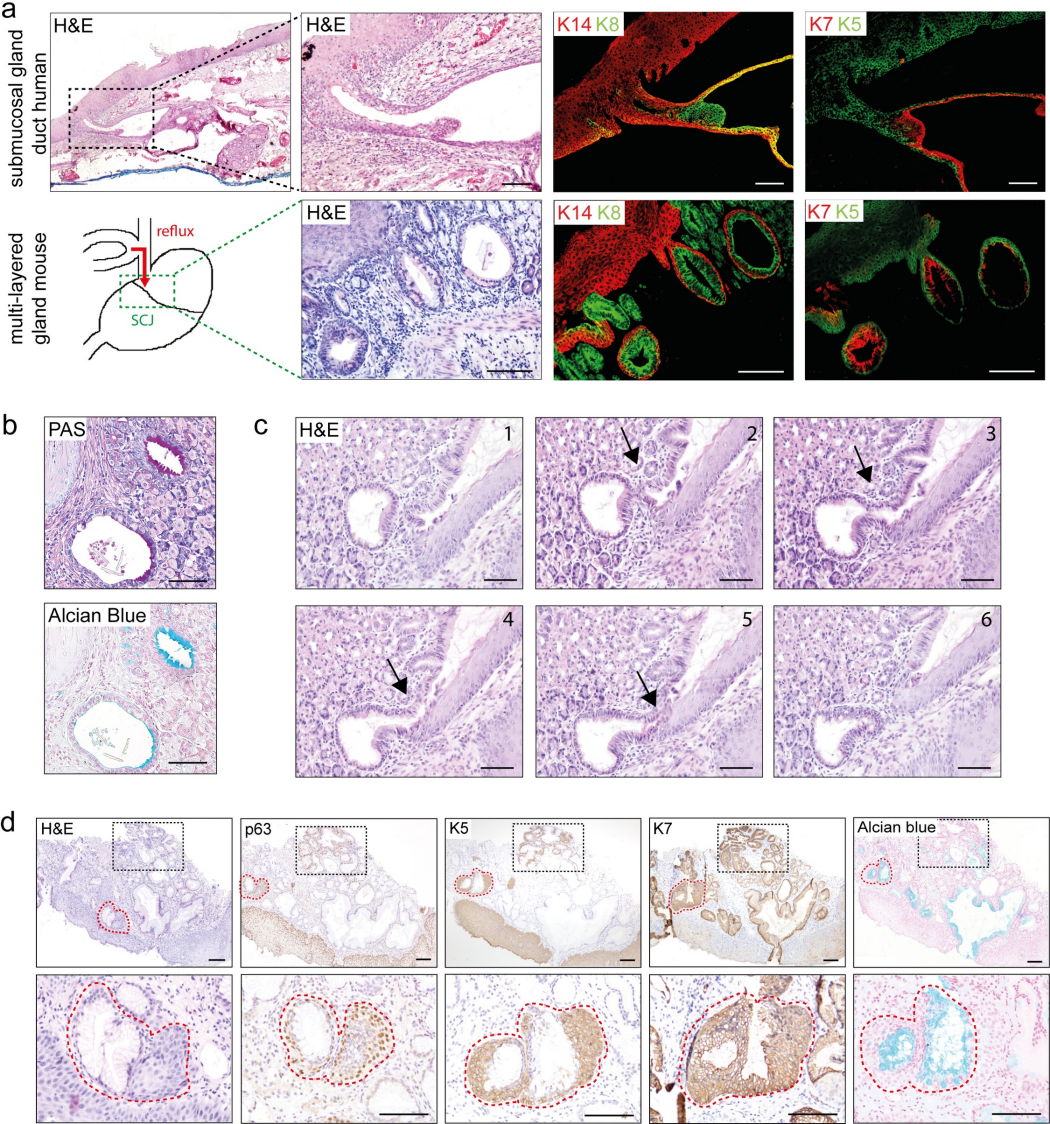
Mouse multi-layered glandular structures (MLGS) show homology to human esophageal submucosal glands ducts (SMGD) and human multi layered epithelium (MLE). [a] Human esophageal submucosal gland (SMG) duct (black dotted box) and mouse MLGS at the SCJ (green dotted box) upon bile stimulation. IF staining for squamous markers K14 (red) and K5 (green) and columnar markers K8 (green) and K7 (red). [b] PAS and Alcian blue staining of multi-layered glandular structures (MLGS) at the SCJ. [c] Serial sections of a MLGS at the SCJ in mice with bile reflux, which extends towards the surface (arrow). [d] IHC for p63, K5, K7 and Alcian blue staining of human early non-goblet cell columnar metaplasia (red dotted line) arising from submucosal multi-layered glandular structures (black dotted box), human esophageal SMGD and murine MLGS. (Scale bars 100μm).

As in these human SMGD, we observed a transition from multi-layered epithelium into stratified squamous epithelium in the MLGS at the SCJ in mice (Fig 1A). The mouse MLGS showed similar cytokeratin (K) expression as the human SMGD, with cells positive for squamous markers K5 and K14 in the outer layer, and for columnar markers K8 and K7 in the inner layer. Of interest is that the population of K7^+^ cells observed in the mice MLGS seem to mark a similar cell population situated on the inner side of the human SMGD (Fig 1A).

We found that the bile reflux induced in these mice increased the mucus production significantly in the MLGS (Fig 1B). Besides secretion of acidic mucins, shown by Alcian Blue staining, secretion of neutral mucins was confirmed by positive periodic acid-Schiff (PAS) staining (Fig 1B). Positive staining for PAS and Alcian blue has also been seen in human SMGD “[22]”.

Additionally, serial sectioning of the MLGS showed that they extended towards the lumen (Fig 1C), suggesting that these glands extend towards the surface to contribute to the mucus producing glandular epithelium.

This seems to be comparable to the early stages of columnar metaplasia development in humans. In a human biopsy, development of non-intestinal columnar epithelium seems to arise from submucosal glands (Fig 1D). This columnar metaplasia shows the same staining as human multi-layered epithelium (MLE), containing both columnar and squamous epithelium “[20]”. The immuno-histochemical and histological features are comparable to that of the mouse MLGS; both structures express the squamous markers p63 and K5 and columnar markers K7 in separate layers (Fig 1D). This similarity between human MLEs in Barrett’s lesions and rodent models has been described earlier “[23]”.

Thus, it seems that surgically induced bile reflux stimulates development of MLGS of mice, which have high homology to the human SMGD. Bile acids can drive the expansion of mice MLGS to develop into a glandular epithelium at the SCJ, which structurally resembles development of columnar metaplasia from the SMGD in humans.

### Human Barrett’s esophagus refluxates mainly contains conjugated bile acids and stimulates expansion of multi-layered glandular structures at the squamo-columnar junction in mice

**Fig 2:**
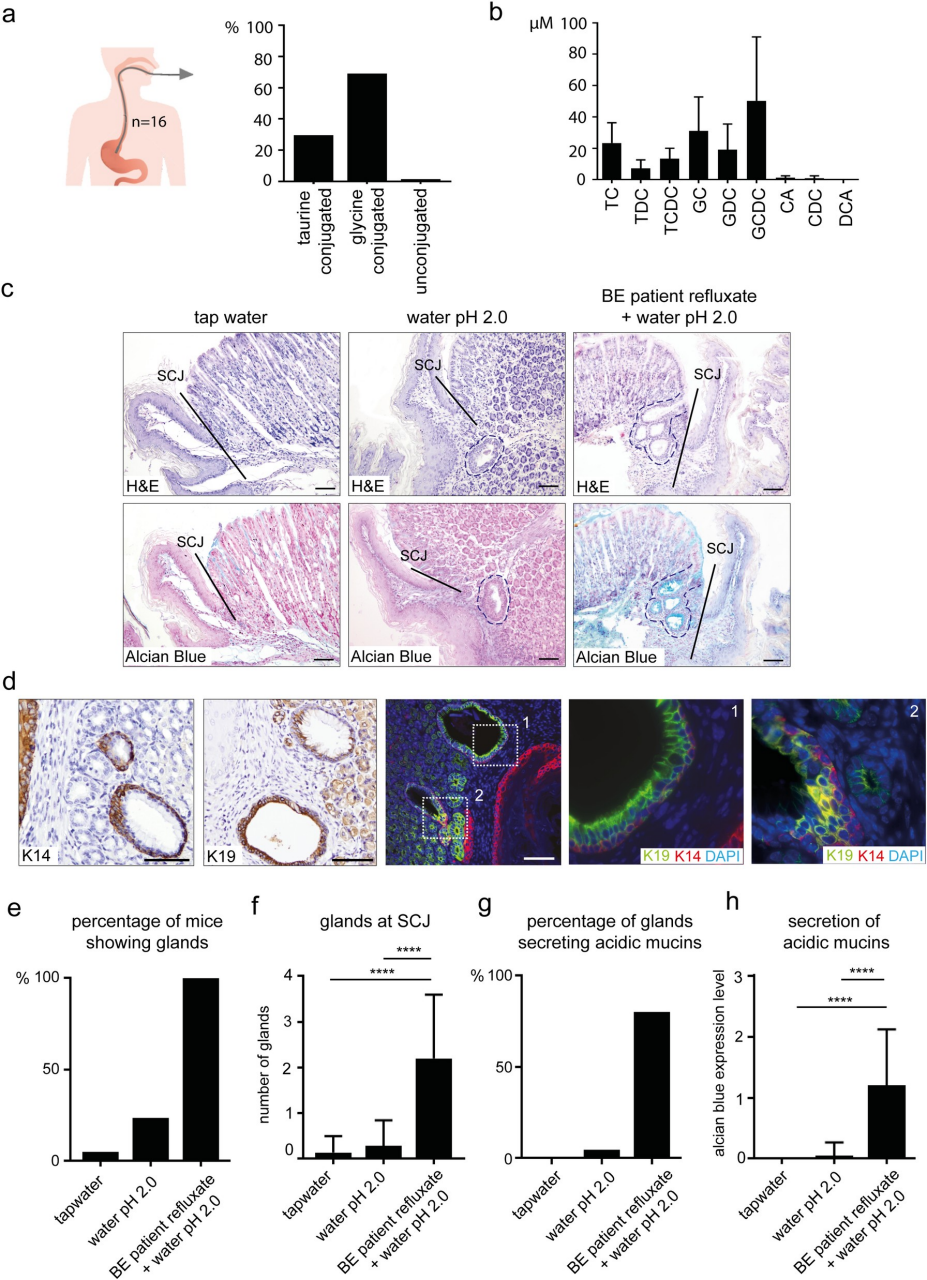
Human BE refluxates mainly contains conjugated bile acids and stimulates expansion of multi-layered gland at the SCJ in mice. [a] HPLC analysis of the bile composition of the refluxates of 16 BE patients. [b] Concentration of bile acids in BE patient’s refluxates determined by HPLC. [c,d,e,f,g and h] Analyses of mice after 8 weeks of treatment with human refluxates via oral gavage combined with acidified drinking water pH2.0 (n = 50), acidified drinking water alone (n = 21), or normal tap water (n = 12). [c] Glands (encircled) at the SCJ (line) in mice after treatment. [d] Single staining for K14 and K19 using IHC and double staining for K14 (red) and K19 (green) using IF of MLGS at the SCJ in mice. DAPI (blue) was used as a nuclear counterstain. [e] Percentage of mice showing glands at their SCJ after treatment. [f] Average number of glands at the SCJ per mouse after treatment. Data are represented as (±SD), Unpaired t test *p<0.05, *p<0.01, ***p<0.001, ****p<0.0001. [g] Percentage of mice showing glands at the SCJ, which secrete acidic mucins after treatment. [h] Secretion of acidic mucins in the glandular structures per mouse after treatment, represented by the expression level of Alcian blue staining (S2C Fig). Data are represented as (±SD), Unpaired t test *p<0.05, *p<0.01, ***p<0.001, ****p<0.0001. (Scale bars 100μm).

To further investigate the role of human bile in development of BE, we analyzed the composition of BA in the refluxates obtained from 16 patients with BE. HPLC analysis showed the presence of taurine (29.7%) and glycine-conjugated BAs (68.9%), with only a low amount of unconjugated BAs (1.4±0.4%) (Fig 2A). This is consistent with the fact that unconjugated BAs are rare in the upper gastro-intestinal tract “[10]”. Further analysis of the bile components showed concentrations (μM) of 23±13 (TC), 7±5 (TDC) and 13±7 (TCDC), 31±22 (GC), 19±16 (GDC) and 50±41 (GCDC) (Fig 2B and S2A Fig).

The effects of bile, with and without acid, have been tested in several animal models, either as chemical components or as an endogenous component in surgical animal models “[11,14,18]”. To test the effects of refluxates from BE patients in wild type mice, BE patients’ refluxates were administrated via oral gavage for 8 weeks in combination with acidified drinking water (pH2.0). Mice on normal tap water or acidified drinking water (pH2.0) were used as controls (Fig 2C). We observed the presence of MLGS at the SCJ. Double immunofluorescence (IF) staining for cytokeratin (K) 14 and 19 showed that the glands comprise of two separate layers (Fig 2D). The outer layer stained positive for squamous markers K5, K14 and p63, while the inner layer stained for the columnar marker K19. The mice MLGS were negative for H^+^/K^+^-ATPase and MUC5AC, indicating that these glands are devoid of parietal and mucus secreting gastric pit cells (Fig 2D and S2B Fig).

The percentage of mice showing glands, as well as the number of glands at the SCJ, increased when acidified drinking water was combined with BAs, compared to acidified drinking water alone (Fig 2E and 2F). The secretion of acidic mucins, represented by Alcian blue staining (S2C Fig) also increased with the addition of BAs. Only 4.8% (1/21) of the mice on acidified water showed glands secreting acidic mucins, while acidic mucin secreting glands were seen in 80% (40/50) of mice when the acidified water was combined with BAs (Fig 2G and 2H).

Together these observations indicate that BAs from BE patients refluxates stimulate development of MLGS at the SCJ in mice, which contain cells carrying either columnar (K19^+^) or squamous epithelial markers (K5^+^, p63^+^).

### Glycine-conjugated bile acids are most effective in inducing the development of multi-layered glandular structures and intestinal type of metaplasia in mice

**Fig 3:**
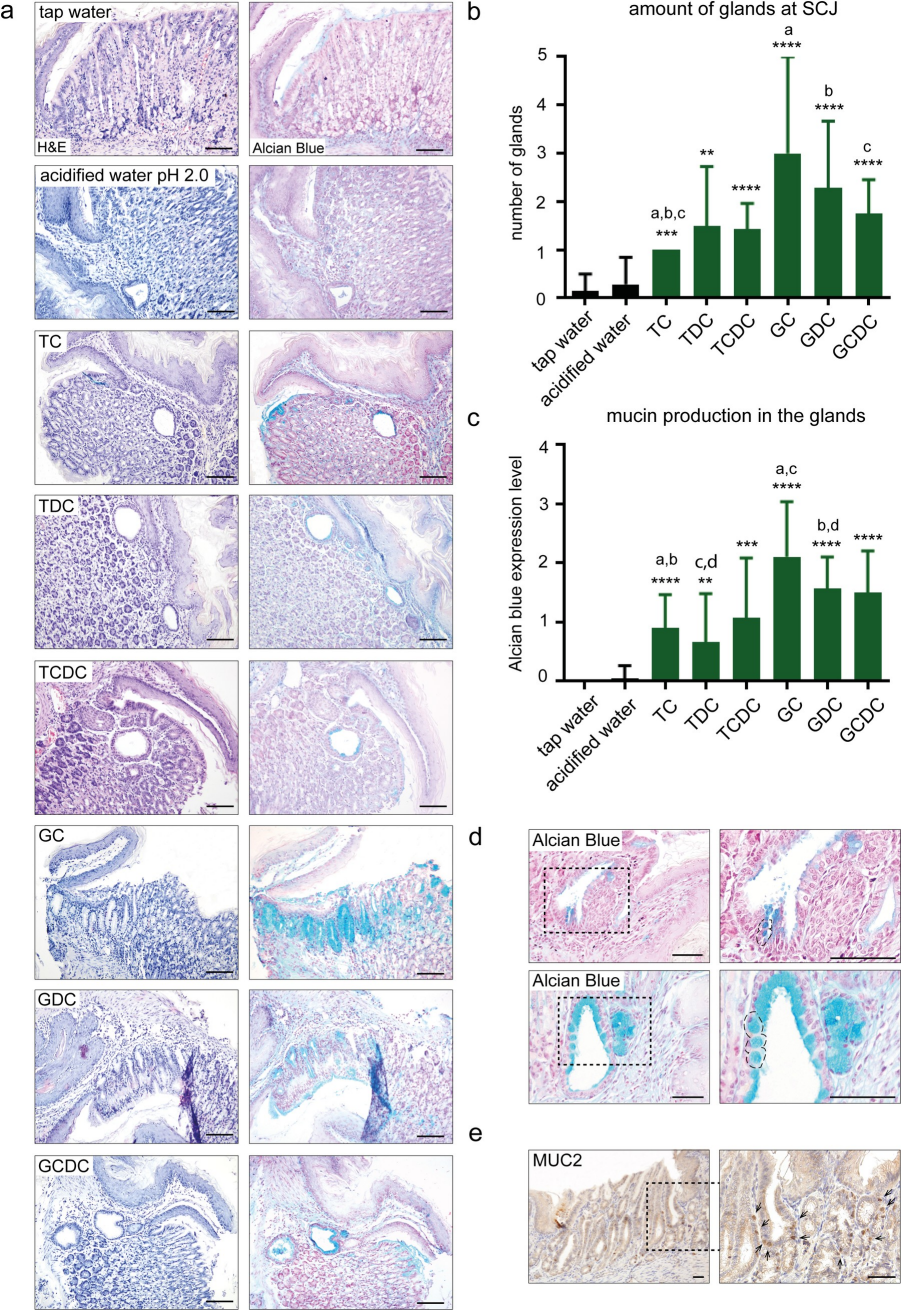
Glycine-conjugated BAs are most effective in inducing the development of multi-layered glandular structures and an intestinal type of metaplasia in mice. [a] H&E and Alcian blue staining of multi-layered glands (MLGS) at the SCJ of mice after 16 weeks of treatment with individual bile components (n = 6 per bile component). [b] Quantification of number of glands and [c] expression level of Alcian blue staining inside the glandular structures (S2C Fig) at the SCJ in mice treated with single BAs for 16 weeks. Treatment with single BAs was compared to treatment with acidified water (pH 2.0). Data are represented as mean±SD. Unpaired t test *p<0.05, *p<0.01, ***p<0.001, ***p<0.0001. Taurocholic (TC), Taurodeoxycholic (TDC), Taurochenodeoxycholic (TCDC), Glycocholic (GC), Glyco-deoxycholic (GDC) and Glycochenodeoxycholic acid (GCDC). [d] Alcian blue staining of MLGS at the SCJ in mice. Goblet cell like structures (encircled) after 16 weeks of glycine-conjugated bile acid treatment. [e] Mucin2 (MUC2) staining of MLGS at the SCJ in mice after treatment with glycine-conjugated bile acids. (Scale bars 100μm).

Glycine-conjugated BAs were the most effective in inducing the development of MLGS in mice (Fig 3A and S3A–S3C Fig). After 16 weeks, both the number of glands and the secretion of acidic mucins in these glands were increased compared to taurine-conjugated BAs (Fig 3B and 3C). Individual components GC, GDC and GCDC showed significantly more glands at their SCJ compared to TC (a, p = 0.0056; b, p = 0.0092; c, p = 0.004). Both GC and GDC also showed significantly more Alcian blue expression compared to TC (a, p = 0.003; b, p = 0.009) and TDC (c, p = 0.027; d, p = 0.035).

Only the glycine conjugated BAs induced goblet cell-like structures (Fig 3D). By using a human/murine specific anti MUC2 antibody “[24]”, which detects early cytoplasmic MUC2 forms, we found perinuclear cytoplasmic staining of a subset of the columnar cells only in mice treated with the glycine-conjugated BA (Fig 3E). MUC2 is a marker for intestinal type of mucins. MUC2 expression was absent in taurine-conjugated BA treated mice (S3E Fig). The intestinal marker caudal type homeobox 2 (CDX2) a nuclear transcription factor for MUC2 in humans, could not be detected in our mice (S3D Fig). Thus, here we show that upon stimulation with glycine conjugated BAs drives the columnar epithelium towards a more intestinal phenotype. Thus, intestinal metaplasia can develop from the MLGS.

### Mice multi-layered glands are a stem cell niche of distinct populations of columnar and squamous stem cells, where K5 positive cells give rise to squamous lineages but not to the columnar epithelium

**Fig 4:**
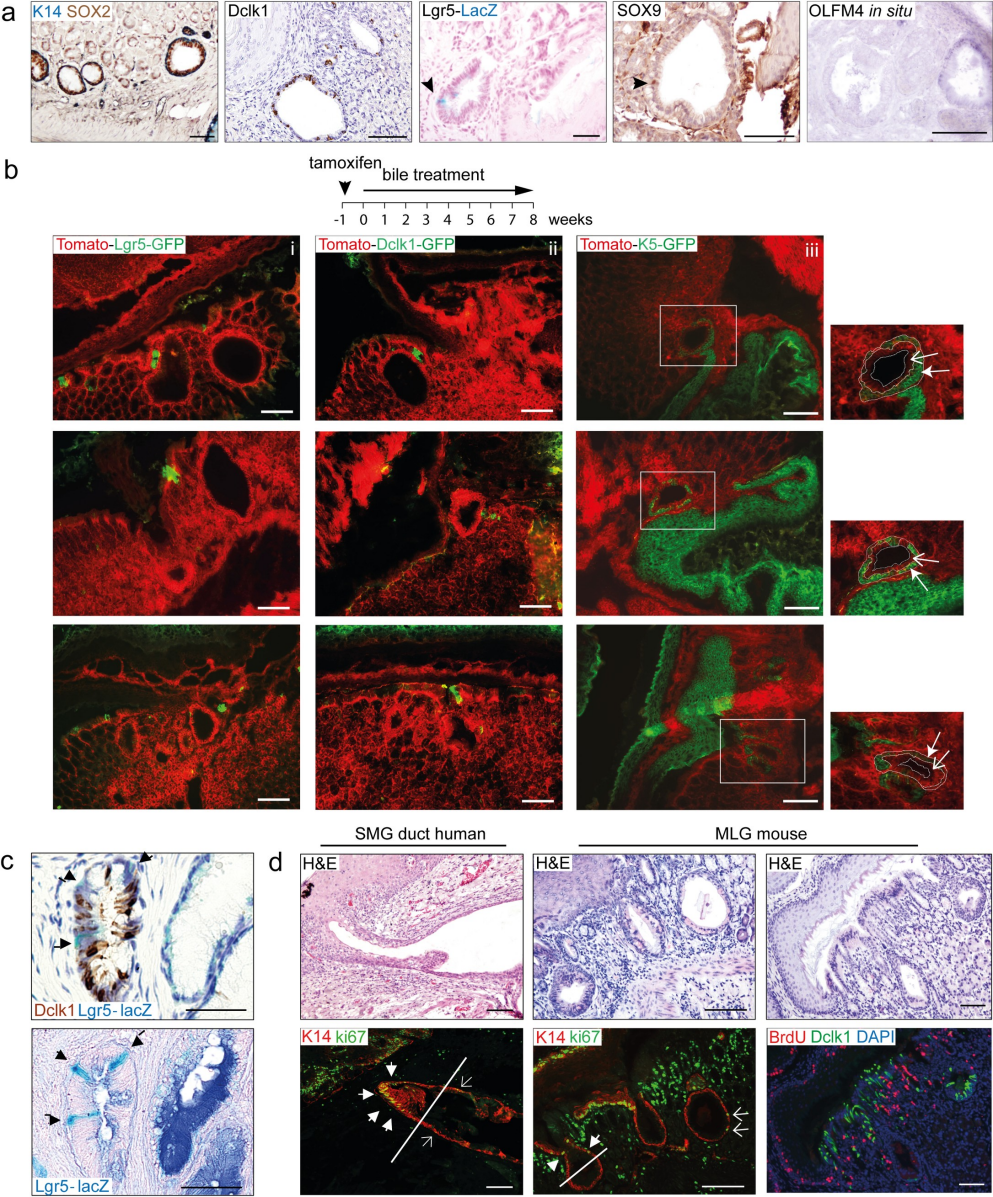
Mice MLGS are a stem cell niche of distinct populations of columnar and squamous stem cells, where K5 positive cells give rise to squamous lineages. [a] Expression of SOX2, K14 (squamous stem cell markers) and DCLK1, Lgr5-lacZ, SOX9 and Olfm4 (*in situ*) (gastric or intestinal stem cell markers) in MLGS.[b] Lineage tracing of Lgr5^+^ cells (i), Dclk1^+^ cells (ii) and K5^+^ cells (iii) after Tamoxifen injection in *Lgr5-cre* (n = 10), *Dclk1-cre* (n = 10) and *K5-cre* mice (n = 10) crossed with *Rosa26-Tomato-GFP* mice. Results of n = 3 mice are shown per cell lineage. All mice were treated with a patient’s refluxate for 8 weeks. Outer squamous (green) layer (closed arrow), inner (red) columnar layer (open arrow). [c] MLGS at the SCJ in mice treated with patient’s refluxate for 8 weeks showing DCLK1 (IHC, brown) and Lgr5-lacZ (blue) positive cells. [d] IF staining for Ki67 (green) and K14 (red) of human SMG ducts and MLGS at the SCJ in mice treated for 8 weeks with patient’s refluxate. Ki67 expression (green), K14 (red) expression in human SMGD and murine MLGS as found at the SCJ. Squamous cells at the upper part towards the luminal surface are indicated by closed arrows. The lower part of the duct and glands are indicated by open arrows. DCLK1 (green) and BrdU (red) expression in the murine MLGS. DAPI (blue) is used as a counterstain. (Scale bars 100μm).

The human SMGD have been suggested to serve as a stem cell niche and as a potential site of origin for BE cells “[25]”. In our mouse model, positive staining for p63, SOX2, K19, Dclk1, SOX9, OLFM4 and Lgr5-lacZ^+^ cells confirmed the presence of squamous, gastric and intestinal progenitors in the MLGS of mice (Fig 4A and S2B Fig) “[26–29]”.

Lgr5 expressing cells have been observed in human BE, suggesting that BE may originate from Lgr5^+^ stem cells “[14,29]”. However, lineage tracing for Lgr5^+^ cells in our model was negative indicating that Lgr5^+^ cells are not involved in the initial expansion of the MLGS (Fig 4Bi and S4A Fig). A similar observation was recently reported by Jiang et al. “[17]”

In intestinal crypts, Dclk1 and Lgr5 do not co-localize and it has been suggested that Dclk1^+^ cells originate from Lgr5-expressing transient amplifying cells “[30]”. In our model, Dclk1 and Lgr5 indeed marked distinct cell populations at the SCJ and in the MLGS (Fig 4C). Hyperplasia, BE metaplasia and tumour development have been found to be accompanied by expansion of Dclk1^+^ cells “[14,27]”. Although we observed a modest expansion of Dclk1^+^ cells within the inner layer of the MLGS (Fig 4A), lineage tracing of the Dclk1 stem cells did not show expansion of these cells in the metaplastic columnar epithelium (Fig 4Bii and S4A Fig).

Also, when we allowed the MLGS to develop first, by administering BAs for 8 weeks, before inducing the lineage tracing at later time points, lineage tracing of Lgr5^+^ and Dclk1^+^ stem cells were still negative (S4B Fig).

We assessed the cellular proliferative status by Ki67 staining and BrdU incorporation to examine if the Dclk1^+^ cells were quiescent. Proliferating cells were detected in the squamous epithelium of the MLGS towards the luminal surface and the overlying squamous tissue. The lower part of the duct and glands had sparse Ki67^+^ cells. Since co-staining of Dclk1 and BrdU was not observed (Fig 4D), the Dclk1^+^ cells seem to be in a quiescent state within the MLGS.

In the recent study by Jiang et al, the ectopic expression of CDX2 in K5^+^ basal cells resulted in expansion of a multi-layered transition zone at the SCJ resulted in intestinal-like metaplasia “[17]”. We performed lineage tracing of K5^+^ basal cells to test whether the columnar epithelium arising from the MLGS in our model using wild type mice are derived from squamous basal cells. Interestingly, BA administration resulted in expansion of K5-GFP^+^ cells in the outer layer of the MLGS, demonstrating that the origin of these cells is from K5^+^ squamous progenitors. However, the inner columnar layer of the gland that expanded into the columnar epithelial layer, was devoid of K5-GFP^+^ cells, suggesting that the origin cell for this layer does not arise from K5^+^ progenitors (Fig 4Biii and S4A and S4B Fig).

Our physiological bile model uses wild type mice and is devoid of genetic modifications. As such our model is a better representation of the human situation. Also, in our surgical model with reflux of endogenous bile “[18]”, lineage tracing of K5^+^ progenitor cells proved to be negative for K5-GFP cells in the metaplastic area at the SCJ, and also in the metaplastic region at the esophago-jejunostomy (S4C Fig).

Unlike the transgenic model of Jiang et al., in our model it seems that during the earliest process of gland expansion and metaplasia development there is a population of cells other than the K5^+^ basal cells that gives rise to inner columnar lining of the MLGS and subsequent columnar metaplasia at the SCJ in our model. Given the negative lineage tracing for several stem cells the mechanism inducing *the initial* expansion of the columnar cells in the MLGS are unclear.

### Glycine-conjugated bile acids cause budding and branching of multi-layered glands followed by crypt fission

The negative Dclk1 and Lgr5 lineage tracing results and the low proliferative activity of the glands indicated that the expansion of the MLGS upon bile stimuli is driven by other mechanisms. One such mechanism is fission, which is started by budding “[31]”. Crypt fission, a process observed in intestinal tissue expansion, is also involved in adenoma growth “[32]”.

**Fig 5:**
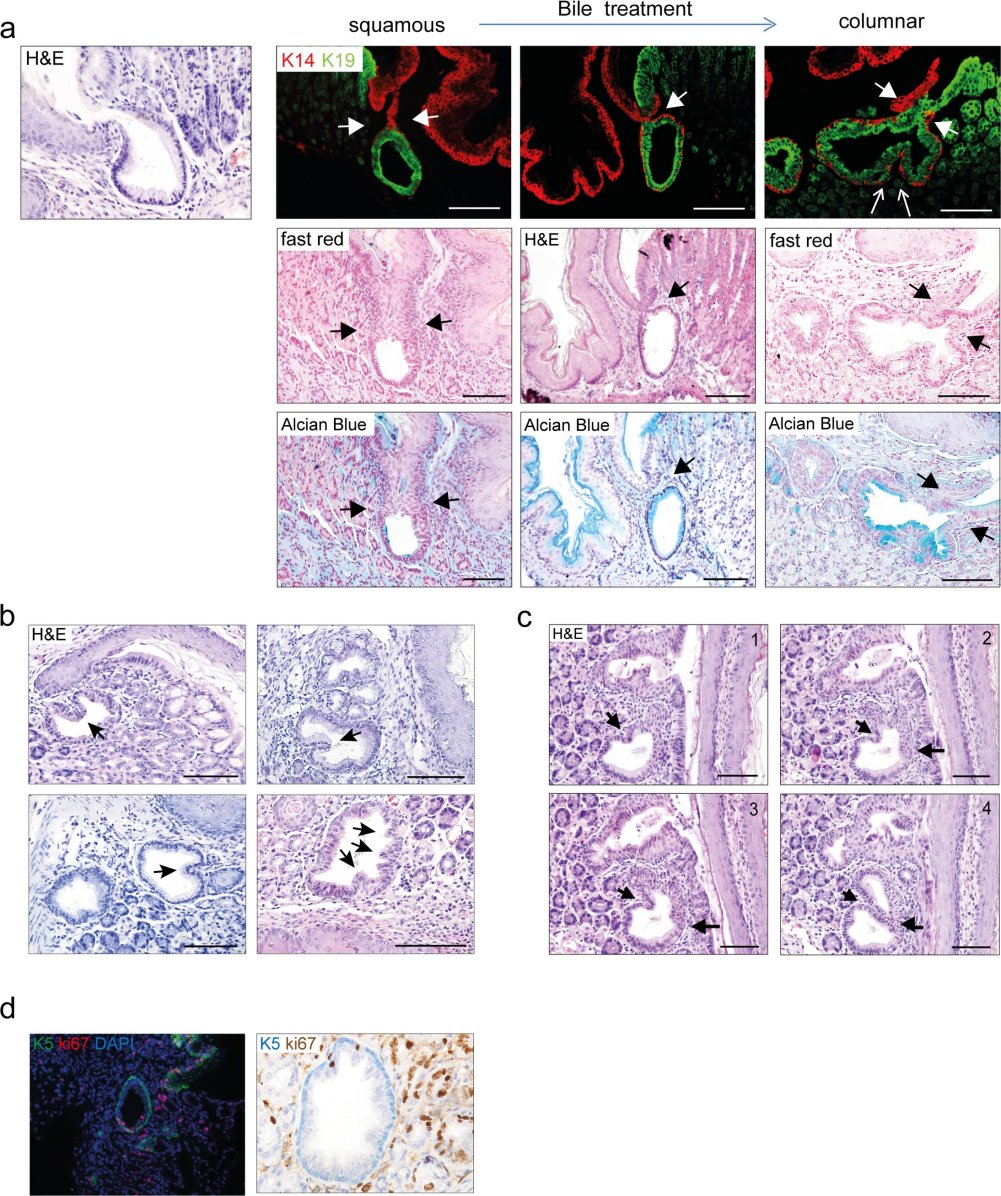
Glycine-conjugated BAs cause budding and branching of MLGS followed by crypt fission. [a] K19^+^ cuboidal cells in the proximal two-thirds (green), and K14^+^ stratified squamous epithelium (red) towards the luminal surface in the MLGS at the SCJ. After glycine-conjugated BA treatment the MLGS extend towards the surface and align with the columnar surface epithelium (closed arrows). Budding of the inner layer after bile treatment (open arrows). [b] H&E staining of MLGS at the SCJ show budding of the inner layer projecting in the lumen of the gland (arrows). [c] Serial sections of the SCJ in mice treated with bile shows budding, which are projecting into the lumen of the MLGS, resulting in the formation of two separate glands by the process of crypt fission. [d] MLGs at the SCJ showing sparse ki67^+^ proliferating cells. (Scale bars 100μm).

We found that a few MLGS spontaneously develop postnatally as during normal development of the mice, which received tap water for 9 months (S2E Fig). In mice treated with the BA the expansion of inner layer columnar of these glands is significantly more pronounced. After 16 weeks of glycine-conjugated BA treatment we found that the inner columnar layer further extends towards the lumen, and gives rise to a single layered mucous producing columnar epithelium, replacing squamous epithelium as observed in the untreated control animals (Fig 5A).

The proliferation of columnar cells resulted in multiplication of columnar cells, followed by the budding of the glands (Fig 5B). A phenomenon that was not seen in mice on tap water (S2E Fig). Serial sectioning of the mice SCJ showed gland budding, resulting in the formation of two separate daughter glands (Fig 5C). The glands have sparse proliferation indicated by a low number of Ki67^+^ cells and typically the proliferating cells appear to be throughout the gland (Fig 5D).

The average number of glands that develop over time upon glycine bile acid treatment was (~5 glands per 16 weeks) 0,38 per week. Together with the low number and the pattern and low number of proliferating cells, this indicates that this initial gland expansion is a relatively slow process and that the expansion is multi-focal and not driven by stem cells from a specific niche.

These results indicate that bile treatment initiates a relatively slow process that eventually leads to multiplication of the MLGS and eventually a columnar lineage. This process is in line with the process of crypt fission. Under normal circumstances crypt fission is a rare event, but it is known to occur more frequently under pathological conditions and in case of certain mutations “[33]”.

## Discussion

Chronic DGERD is a strong risk factor that correlates with BE development and progression to EAC “[2,9]”. Here, we demonstrate which bile acids are most potent for inducing development of metaplastic glands and the initial mechanism underlying this process at the squamo-columnar junction in a mouse model.

The detergent behavior of BAs depends on concentration, the pH of the solution and the pKa value of individual BA species ‘[34]”. When the pH is lowered towards these pKa values, BAs are protonated, making them less soluble and able to enter the epithelial cells via passive diffusion. After entering the cell, the acid is ionized because of the neutral pH and becomes trapped, where they can affect important pathways. Before BE is diagnosed and before PPIs are prescribed to relieve symptoms, the refluxate of patients will be still acidic. In such an acidic environment, especially the taurine-conjugated BAs, which we found to be high in our BE patients, are highly damaging and harmful “[34]”.

Under more neutral pH (PPI use results in increased pH (6.3±0.8, S2F Fig), we demonstrate that in particular the glycine-conjugated BAs are able to induce an inflammatory response and development of intestinal type of metaplasia. Esophageal epithelial cells do not express the hepatic bile acid uptake transport protein NTCP and thus transport of bile acids from lumen into esophageal squamous cells entirely depends on passive diffusion. In contrast to taurine-conjugated bile acids, glycine-conjugated bile acids in an acidic milieu are uncharged and fully protonated, which enables them to cross membranes “[35]”. This may be the reason why glycine-conjugated bile acids are more effective in inducing damage than the negatively charged and membrane-impermeable taurine-conjugates in this model. We presume that this damage leads to the release of cytokines causing inflammation, and growth factors that during repair induces replacement of the damaged epithelium by the more bile acid resistant metaplastic cells.

The immunohistochemistry of the MLGS, observed at the SCJ in mice that were treated with the refluxates, showed striking similarities with the human esophageal SMGD. Our observations contrast with a previous study suggesting that mice lack submucosal structures that resemble the human SMG rendering mouse models as not suitable for studying reflux disease “[16]”. We show that MLGS are comparable to the SMGD in their double-layered structure with a matching cytokeratin expression profile. Moreover, they structurally share the opening towards the lumen “[21]” and like the SMGD have the ability to respond to damage by inducing mucus production and an expansion of the number of Dclk1^+^ cells “[14,27]”. Human SMGD are thought to be the source of BE metaplasia and subsequently EAC “[36]”. The mouse MLGS seem to represent the ductal part of the human MLG and may serve as a model of metaplasia arising from the MLGD. This suggests that at least in part, the results from this model can be translated to humans. Our observations are in line with a more recent by Jiang *et al*, who describes a multi-layered epithelium at the SCJ in mice “[17]”.

Currently, it is not clear which stem cells give rise to, and if stem cells are at the basis of the initial development of metaplastic glands as observed in BE. Glickman “[20]” showed that SMGD have a similar phenotypic pattern as compared to BE and, can give rise to multi-layered epithelium (MLE), which has been suggested to represent an early stage of columnar metaplasia. The fact that SMGD are not only associated with squamous epithelium “[22]”, but also with BE “[25]” and neo-squamous tissue within BE tissue “[37,38]” suggests the presence of pluripotent stem cells or multiple cell lineages in the SMGD. The MLGS observed in our mouse model, showed a unique compendium of lineages “[39]”, including the presence of p63^+^ and SOX2 squamous (stem) cells, but also CK19^+^ columnar cells “[26]” and Dclk1 “[27]”, Lgr5 “[40]”, SOX9 “[41]” and OLFM4 “[28]” positive intestinal stem cells. The clinical significance of the identification of a murine gland duct stem cell niche, the MLGS, which highly resembles the human SMGD, is therefore of high importance.

We have shown that exposure to conjugated BAs found in the refluxates from BE patients resulted in MLGS at the SCJ in mice. Although in the past several hypotheses have been proposed to identify the site of origin of the metaplasia, more recent murine studies point to the presence of either dedicated or multi-potent stem cells situated either as embryonic remnants “[15]” or in multi-layered epithelium at the SCJ. The multilayered epithelium was recently indicated by Jiang et al “[17]”, which they compared to human MLE found by Glickman et al. “[20]”. They suggest that K5^+^p63^+^K7^+^ basal cells serve as progenitors for intestinal-like epithelium “[17]”. However, this effect was only seen in a transgenic model with ectopic expression of CDX2 in K5^+^ basal cells.

In contrast, in our model using wild type mice, the intestinal metaplasia that developed was negative when lineage traced for K5^+^ cells was performed, suggesting that the K5^+^ cells from the basal/outer layer of the MLGS do not give rise to the columnar metaplasia as observed in our model. The intestinal metaplasia in our model, expressed MUC2 but not its putative transcription factor CDX2. This means that in our model the MUC2 transcription is probably regulated through alternative routes. Indeed, in mice MUC2 expression has been found to be regulated by GATA4, which is frequently expressed in goblet cells “[42]”. We believe that the pathophysiological effects of bile acids as can be found in DGERD and BE patients are more closely mimicked in our model, and that our findings are more translatable to the human situation.

We observed budding of the inner columnar lining of the cells and eventually doubling of the glands, which suits the process of crypt fission. Development of glands i.e. mammary glands and tracheal SMGs, is initiated during embryonic development, but develops further during postnatal life via branching morphogenesis “[43]”. Branching morphogenesis is a complex developmental process, which involves activation of BMP4 “[44]”. Based on our previous findings “[18,45]”, we believe that re-activation of the BMP4 pathway, upon bile stimulation “[46]”, most likely causes the MLGS to undergo branching and crypt fission. Crypt fission has been observed for instance in normal adult intestinal epithelium. Under healthy conditions crypt fission is reported to occur only once in 30 years in colon crypts “[47]”. However, this process can be re-activated in case of mucosal damage and for instance drives the clonal expansion of mutant crypts in adenomas “[48]”.

The involvement of stem cells in the process of fission is unclear. Despite negative lineage tracing for K5^+^, Dclk1^+^ and Lgr5^+^ cells, stimulation of glands with BAs consistently resulted in an increased number of MLGS. Together, with the low frequency of BrdU incorporation and low number of KI-67^+^ proliferating cells, we presume that *initial* expansion of the MLGS may not depend on stem cell proliferation but on the involvement of other cellular processes affecting cellular plasticity. More recently the process of ‘paligenosis’ has been described, during which mature cells return to a regenerative stage “[49]”. Cellular dormancy which has been recognized in cancer progression and involves the TGF-beta/BMP pathway, could be another process leading to the reactivation of dormant cells within the MLGS “[50]”.

Future experiments using this refluxate model are necessary to further characterize the molecular processes underlying the initial processes leading to columnar metaplastic epithelium at the SCJ, and to translate these findings to the human Barrett’s esophagus.

In summary, the observation of mouse MLGS resembling the human SMGD indicates that these structures are valuable to study the pathogenesis of metaplasia in mouse models. We showed that, glycine-conjugated BAs are able to induce glandular metaplasia through the process of crypt fission, eventually leading to an intestinal type of metaplasia. We indicate the presence of both squamous and columnar cell lineages in the MLGS and in this physiological BA model excluded the possibility that the metaplastic columnar epithelium from the MLGS is derived from K5^+^ squamous progenitor cells, but also from Dclk1 and Lgr5^+^ columnar progenitors, and we speculate that the initial process of gland expansion is independent of stem cell proliferation. These novel insights into the structural events provoked by specific BAs, indicate that future strategies for more effective prevention of BE and EAC development should target different refluxate components including BAs. Moreover, having a much more physiologically relevant Barrett model at hand for studying the development of IM arising from SMGD-like structures, is of major importance to further delineate the molecular events underlying the metaplasia and subsequent carcinogenesis process.

## Materials and methods

### Human biopsy specimens, bile sampling and analysis of human refluxates by HPLC

Human research using patient material was approved by the institutional ethical committee of the Academic Medical Center of Amsterdam (AMC). All patients received informed consent and gave written permission to participate in the study. Routine endoscopic biopsies of BE patients, on PPI, taken during surveillance endoscopy. Biopsies from patients with early non-goblet cell columnar metaplasia were stained for columnar and squamous markers and compared to the murine multilayered glandular structures. From patients with confirmed BE, without dysplasia, refluxates from the proximal stomach were sampled by using a suction catheter during endoscopy between March 2016 and January 2017. Samples were stored at -180 degree Celsius until analysis or administration to the animals. From 16 of these patients the biliary composition was determined by reverse-phase high-performance liquid chromatography as earlier described “[51]”.

### Bile components for bile mixture

Taurocholic acid, Taurodeoxycholic-, Taurochenodeoxycholic-, Glycocholic-, Glycodeoxycholic- and Glycochenodeoxycholic acid were all purchased from Sigma-Aldrich. The bile acids stock solutions were prepared in sterile water for injection. The six conjugated bile components were mixed in the same ratio as found in the human refluxates: TC 15,9%, TDC 4,9%, TCDC 9,0%, GC 21,2%, GDC 13,0%, GCDC 34,3% (S2D Fig). After preparation the mixture of bile acids was filtered through 0.2 μm Acrodisc Syrenge filter (Pall) and stored at 4°C. Each individual bile component was used at a concentration of 10mM.

### Surgical mouse model

Twelve CB6F1 male mice were used after formal approval of the Animal Experimental Committee (DEC) of the Amsterdam Medical Center in compliance with the Animal Welfare Body (IvD) protocol LEX102475. All animals were kept in the Animal Research Institute of the AMC (ARIA), and experiments were performed under ARIA standard operating procedures (SOP).

Mice were socially housed in +/+ IVC cages in ventilated racks with tissues as environmental enrichment. Experiments were performed using 8–10 week old male mice, with initial weight above 20g. After the procedure animals received liquid food for 2 days. Afterwards, normal pellet food and tap water were available *ad libitum*.

Mice were subjected to inhalation of 2% isoflurane for induction and maintenance of anesthesia. The animals were gently fixed to a heating pad (37°C) and after weighing the abdomen shaved and cleaned with a 2% (v/v) chlorhexidine gluconate/70% (v/v) isopropyl alcohol solution. A single injection of pain relieve (5mg/kg, Melovem) was administrated subcutaneously. In these animals an anastomosis between the esophagus and jejunum was created by implanting a 1.58 x 0.78 mm neodymium micro magnets to provide pressure necrosis between the jejunum and esophagus to obtain a fistula to induce reflux of bile into the distal esophagus. The first magnet was placed in the lower half of the esophagus and a second magnet was placed via a separate jejunostomy in a post ligament of Treitz jejunal loop (S1A Fig). The animal were kept on a warming blanket for the first 24 postoperative hours. Animals were weighted every day during the first week, followed by twice in a week. Animals were euthanized immediately in case of severe weight loss, severe dehydration, stress at any point after surgery. Within several days the approximated magnets cause an esophago-jejunal fistula, while the magnets are excreted via the stools. Animals were sacrificed 6, 12, and 16 weeks after the operation or when humane endpoints were reached. Human endpoints were reached when animals present swallowing complications/dysphagia and show no improvement after 72 hours, when animals present weight loss to a maximum of 15% compared to the start of the experiment or extended period of weight loss or if the animal lapses into a poor state in any other way. In case humane endpoints were reached, mice were euthanized within 24 hours. All mice (n = 12) were euthanized at the end of the experiment. Euthanasia was performed by induction with CO2/O2 (40/60) until animals were rendered unconscious at which point the CO2 was increased to 100% until the animal was dead. Tissues of the anastomosis site and the SCJ were formalin fixed and paraffin embedded for IHC “[18]”.

### Refluxate mouse model

All mice were used after institutional approval of the animal ethical committee of the study (Academic Medical Center, LEX102612). Mice were socially housed in +/+ IVC cages in ventilated racks with tissues as environmental enrichment and normal pellet food and tap water available *ad libitum*. Experiments were performed using 8–10 week old male mice, with initial weight above 20g. CB6F1 male mice were purchased from Janvier (France). Mice were treated with refluxates from BE patients (n = 50) (Fig 2C) or individual BAs (Fig 3A, S3A Fig, n = 6 per component, per time point). Biles were administered via oral gavage, 250μL per day, 5 days a week for 8 or 16 weeks. After given oral gavage, drinking water was taken away for 1,5 hour. Mice were either on normal tap water (n = 12) or acidified drinking water (pH2.0) (combined with bile n = 50, without bile n = 21). Animals were weighted every day during the whole experiment and checked for signs of weight loss. Mice that suffer from weight loss of show signs of discomfort will be taken of bile trea^tm^ent until they are free of discomfort and regained their weight. Animals were sacrificed 8 or 16 weeks after the start of the experiment or when humane endpoints were reached. Human endpoints were reached when animals present weight loss to a maximum of 15% compared to the start of the experiment or extended period of weight loss or if the animal lapses into a poor state in any other way. In case humane endpoints were reached, mice were euthanized within 24 hours. All mice were euthanized at the end of the experiment. Euthanasia was performed by induction with CO2/O2 (40/60) until animals were rendered unconscious at which point the CO2 was increased to 100% until the animal was dead. Stomach tissues were formalin fixed and paraffin embedded for H&E and Alcian blue staining and for IHC.

### Quantification of multi-layered glandular structures and mucus secretion at the squamo-columnar junction in mice

Hematoxylin and eosin (H&E) and Alcian blue staining were performed on all tissues. The number of mice that showed MLGS at their SCJ were counted and represented as “percentage of mice showing glands”. The “number of glands” per mouse was counted over 20 consecutive slides and the highest number was taken forward.

The number of mice that showed glands with positive Alcian blue staining were counted and represented as “percentage of mice secreting acidic mucins”. The “secretion of acidic mucins” was measured semi-quantitively, taking into account the intensity and amount of blue staining of the glands: 0 = no blue staining (0%), 1 = low blue staining (~10%), 2 = medium blue staining (~10–50%) and 3 = high blue staining (>50%). All the analyses were performed blinded from the treatment that mice received.

### Immunohistochemistry + Immunofluorescence

Tissue slides were de-paraffinized and antigen retrieval was performed in citrate buffer pH 6 for 20 min at 98 degrees. Slides were blocked in 3% H_2_O_2_ for 30 minutes followed by 10% goat serum for 30 min. Slides were incubated with primary antibodies (Table 1). Slides were washed in TBS + 1%Tween 20 and incubated with either the respective biotin linked secondary reagents from the LSAB Kits (Dako, Belgium) following the manufacturer’s instructions for IHC or with goat-anti-rabbit-fluor488 or goat-anti-mouse-fluor568 for IF. Peroxidase activity was visualized using DAB+ (Dako, Belgium). IHC sections were counterstained with Mayer’s haematoxylin, dehydrated and mounted. IF glass slides were mounted with DAPI (Roche, Mannheim, Germany)/vectashield (Vector laboratories Inc, Burlingame, CA, USA). Images were acquired on an Olympus BX51 fluorescent microscope using cell^F software (Olympus Optical, Tokyo, Japan).

**Table 1:** Antibodies used for IHC and IF.

P63	1:100	Santa cruz	H^+^K^+^	1:100	MBL
K5	1:150	Epitomics	SOX9	1:500	Abcam
K14	1:100	Epitomics	MUC2	1:1000	Gift from Rotterdam
K8	1:100	Epitomics	MUC5AC	1:100	Thermo Scientific
SOX2	1:100	Epitomics	K7	1:10	Progen
CDX2	1:100	Epitomics	Ki67	1:100	Thermo Scientific
K19	1:100	Epitomics	BrdU	1:75	Abcam
Dclk1	1:100	Epitomics			

Lgr5^+^ cells were visualized by β-galactosidase staining in *Lgr5- creERT* mice “[29,40]” crossed with *rosa26-lacZ mice*. Tamoxifen (3 days, 1mg) was injected 7 days before sacrificing (Table 2).

**Table 2:** Tamoxifen concentrations used for lineage tracing in mouse models.

K5-cre x Rosa26-Tomato-GFP	0.25mg	3 consecutive days
Dclk1-cre x Rosa26-Tomato-GFP	1mg	3 consecutive days
Lgr5-cre x Rosa26-Tomato-GFP	1mg	3 consecutive days
Lgr5-cre x Rosa26-lacZ	1mg	3 consecutive days

### In situ hybridization

In situ hybridization for OLFM4 was carried out as described previously “[29]”.

### Lineage tracing

*Lgr5-creERT “*[29,40]” and *Rosa26-Tomato-GFP* were purchased from Jackson, USA. *K5-creERT* mice were a kind gift from CM Chen, Taiwan “[52]”. *Dclk1-creERT* mice were a kind gift from H. Seno, Japan “[30]”. All mice were used after institutional approval of the animal ethical committee of the study (Protocol 102475 and 102840). *K5-cre* (= 25), *Lgr5-cre* (n = 25) and *Dclk1-cre* (n = 25) mice were crossed with *Rosa-Tomato-GFP* mice and injected with Tamoxifen (Table 2) to induce lineage tracing. Mice were injected with Tamoxifen for 3 days and sacrificed after 7 days to see the expression in normal esophageal, SCJ and intestinal tissue (n = 5 per gene). Mice were injected with tamoxifen followed by bile treatment via oral gavage for 8 weeks (n = 10 per gene) (Fig 4B). Another group of mice was treated with bile for 8 weeks, before tamoxifen injection, followed by another 8 weeks of bile treatment (n = 10 per gene) (S4B Fig).

Animals were weighted every day during the whole experiment and checked for signs of weight loss. Mice that suffer from weight loss of show signs of discomfort were taken of bile treatment until they were free of discomfort and regained their weight. Animals were sacrificed 7 days, 8 weeks or 16 weeks after the start of the experiment or when humane endpoints were reached. Human endpoints were reached when animals present weight loss to a maximum of 15% compared to the start of the experiment or extended period of weight loss or if the animal lapses into a poor state in any other way. In case humane endpoints were reached, mice were euthanized within 24 hours. All mice were euthanized at the end of the experiment. Euthanasia was performed by induction with CO2/O2 (40/60) until animals were rendered unconscious at which point the CO2 was increased to 100% until the animal was dead. Stomach tissues were formalin fixed and paraffin embedded for H&E and Alcian blue staining and for IHC.

### Statistics

Unpaired t-test were performed in GraphPad to test statistical differences in number of glands and Alcian blue expression levels (secretion of acidic mucins) between mice treated with human refluxates compared to drinking water pH2.0 alone (tap water are comparable results). Statistical significant differences were also tested in between groups (a,b,c). Statistical significance was set at p < 0.05. Unpaired t-test: *p<0.05, **p<0.01, ***p<0.001, ****p<0.0001.

## Supporting information

S1 Fig Effect of surgically induced reflux of acid and bile. [A] Surgically induced reflux of acid and bile by creating an esophago-jejunostomy “[18,19]”. The model allows reflux of acid and bile from the intestine into the esophagus and stomach (red arrow). [B] H&E and Alcian Blue staining of anastomotic site (blue dotted box) after 6 (n = 4), 12 (n = 4) and 16 (n = 4) weeks after surgically inducing reflux. Eso, esophagus (red dotted line) and metaplastic area (purple dotted line). [C] H&E and Alcian Blue staining of squamo-columnar junction (SCJ) in mice stomach (green dotted box), 6, 12 and 16 weeks after surgically inducing reflux. Scale bars 100μm.
(TIF)

S2 Fig Effect of treatment with human bile in mice. [A] Percentage of each individual bile component found in BE patients refluxates as analyzed by high performance liquid chromatography (HPLC) “[51]”. [B] IHC for squamous markers p63 and K5 and columnar markers H^+^K^+^ (parietal cells) and MUC5AC (gastric pit cells) in multilayered glandular structures (MLGS) at the SCJ in mice after treatment with BE patients refluxates for 8 weeks (n = 50). [C] Alcian blue expression level, representing the secretion of acidic mucins, in the MLGS at the SCJ in mice after bile treatment. 0 = Alcian blue absent, no secretion of acidic mucins; 3 = high levels of Alcian blue staining inside the MLGS representing massive acidic mucin secretion. [D] The 6 individual conjugated BAs were purchased from Sigma and mixed together in the same ratio (TC:TDC:TCDC:GC:GDC:GCDC, 3:1:2:4:3:7) as found in human refluxates. Oral gavage of the sigma mix (pH ~7) was as effective as the human refluxate samples in inducing gland development at the SCJ in (6/6) mice, in both number of glandular structures and mucin production. [E] Development of MLGS at the SCJ in mice after 9 months on normal tap water and acidified drinking water pH2.0. [F] pH of human refluxates with (6.3±0.8) or without (2.0±0.8) taking PPIs. Scale bars 100μm.
(TIF)

S3 Fig Effect of individual bile components in mice. [A] H&E and Alcian blue staining of the SCJ in mice treated with individual bile components (10mM) for 8 weeks resulting in the development of MLGS (n = 6 per bile component). [B] Quantification of number of glandular structures and [C] the secretion of acidic mucins, represented by the amount of Alcian blue staining inside the glandular structures (S2C Fig), at the SCJ in mice treated with individual BAs for 8 weeks. Treatment with individual bile components was compared to treatment with acidified water pH 2.0. Data are represented as mean±SD. Unpaired t test *p<0.05, *p<0.01, ***p<0.001. Individual components GC showed significant more glandular structures at their SCJ compared to TC, TDC and TCDC (a, p = 0.009; b, p = 0.038; c, p = 0.047). No differences were seen in Alcian Blue expression inside the glands. Taurocholic (TC), Taurodeoxycholic (TDC), Taurochenodeoxycholic (TCDC), Glycocholic (GC), Glyco-deoxycholic (GDC) and Glycochenodeoxycholic acid (GCDC). [D] IHC for intestinal marker CDX2 in MLGS at the SCJ in mice. [E] IHC for intestinal marker MUC2 in MLGS at the SCJ in mice treated with taurine conjugated bile acid. Scale bars 100μm.
(TIF)

S4 Fig Lineage tracing of intestinal Lgr5, Dclk1 and squamous K5 cells following bile treatment. [A] Expression of Lgr5-GFP, Dclk1-GFP and K5-GFP positive cells in the normal esophagus, at the normal SCJ and in the intestine: *Lgr5-cre* (n = 5) “[29]”, *Dclk1-cre* (n = 5) “[27]” and *K5-cre* (n = 5) “[52]” mice crossed with *Rosa26-Tomato-GFP* mice were injected with Tamoxifen for 3 days and sacrificed after 7 days to see the expression in normal esophageal, SCJ and intestinal tissue. [B] Expression of K5-GFP, Dclk1-GFP (white arrows) and Lgr5-GFP (white double arrows) positive cells in MLGS at the SCJ in mice (white dotted areas) after: *Lgr5-*c*re-Tomato-GFP* (n = 10), *Dclk1-cre-Tomato-GFP* (n = 10) and *K5-cre-Tomato-GFP* (n = 10), mice were treated with BE patients refluxate for 8 weeks to allow development and multiplication of the MLGS at the SCJ. After 8 weeks, mice were injected with tamoxifen for 3 days and treated with bile acids for another 8 weeks. [C] Tamoxifen injection (3 days, 0,25mg i.p.) followed by surgically induced reflux resulted in metaplastic area (purple or white dotted line) at the anastomotic site which was negative for K5-GFP positive cells. The squamous tissue, eso (esophagus, red dotted area) was positive for K5-GFP positive cells. Scale bars 100μm.
(TIF)

## Abbreviations

AB: Alcian Blue
BA: Bile acid
BE: Barrett’s esophagus
b-gal: Beta-galactosidase
CA: Cholic acid
CDCA: Chenodeoxycholic acid
CDX2: Caudal type homeobox 2
Dclk1: Doublecortin-like kinase 1
DGERD: duodenal-gastro-esophageal reflux disease
EAC: Esophageal adenocarcinoma
GERD: Gastro-esophageal reflux disease
GC: Glycocholic acid
GCDC: Glycochenodeoxycholic acid
GDC: Glycodeoxycholix acid
GFP: Green fluorescent protein
HPLC: high-performance liquid chromatography
IHC: Immunohistochemistry
IF: Immunofluorescence
K: Cytokeratin
Lgr5: Leucine-rich-repeat-containing G-protein-coupled receptor 5
MLE: Multilayered epithelium
MLGS: multi-layered glandular structures
OLFM4: Olfactomedin 4
PAS: Periodic acid-schiff
PPI: Proton pump inhibitor
SCJ: Squamo-columnar junction
SMG: Submucosal gland(s)
SMGD: Submucosal gland duct(s)
SOX: SRY (sex determining region Y)-box
TC: Taurocholic acid
TCDC: Taurochenodeoxycholic acid
TDC: Taurodeoxycholic acid
