# Supplementary figures and images for: Glyco-conjugated bile acids drive the initial metaplastic gland formation from multi-layered glands through crypt-fission in a murine model
Source: PLoS One. 2019 Jul 26;14(7):e0220050. doi: 10.1371/journal.pone.0220050 (PMC6660124; doi:10.1371/journal.pone.0220050)

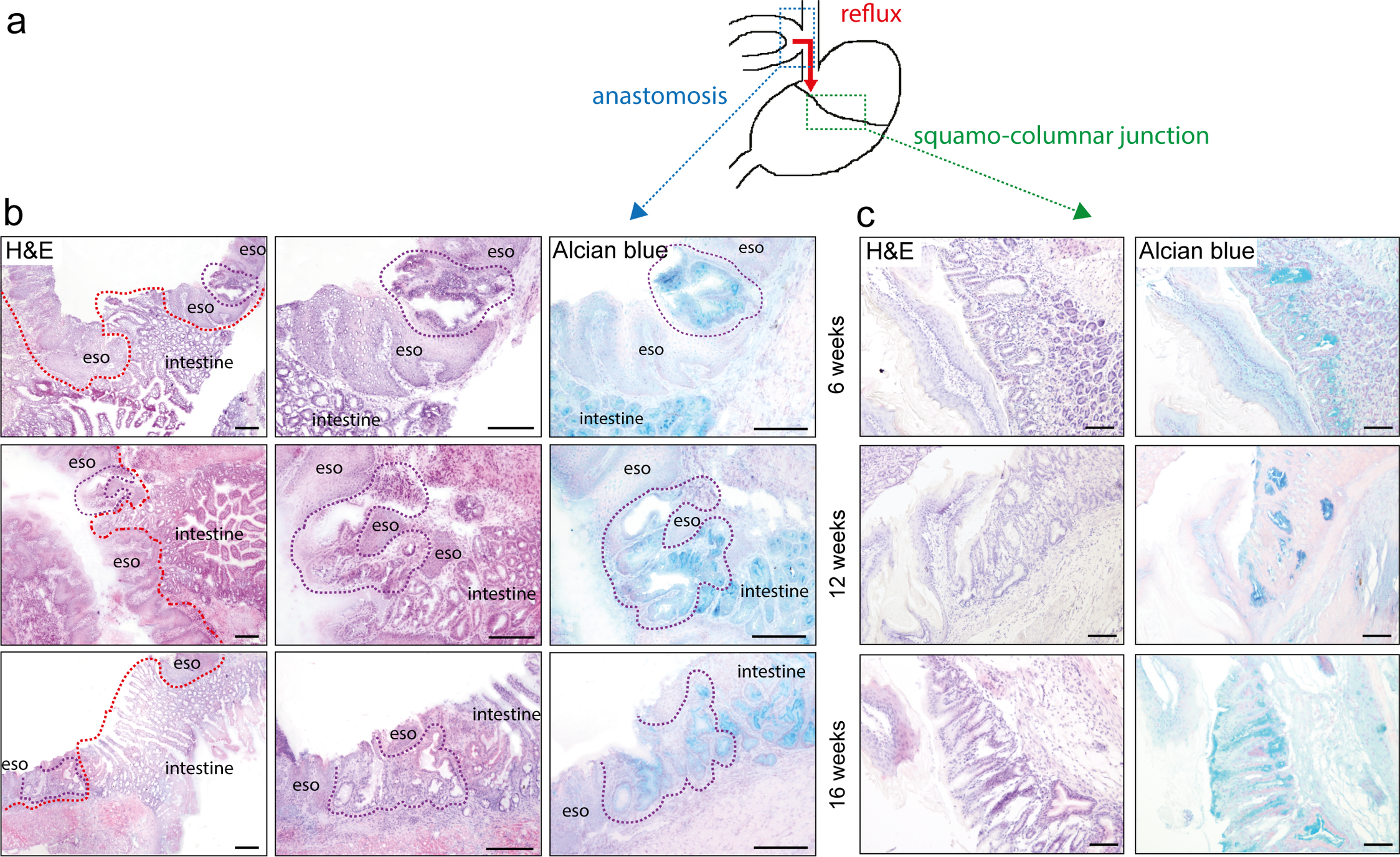

Supplement: S1 Fig — [A] Surgically induced reflux of acid and bile by creating an esophago-jejunostomy “[18,19]”. The model allows reflux of acid and bile from the intestine into the esophagus and stomach (red arrow). [B] H&E and Alcian Blue staining of anastomotic site (blue dotted box) after 6 (n = 4), 12 (n = 4) and 16 (n = 4) weeks after surgically inducing reflux. Eso, esophagus (red dotted line) and metaplastic area (purple dotted line). [C] H&E and Alcian Blue staining of squamo-columnar junction (SCJ) in mice stomach (green dotted box), 6, 12 and 16 weeks after surgically inducing reflux. Scale bars 100μm. (TIF) [file pone.0220050.s001.tif]

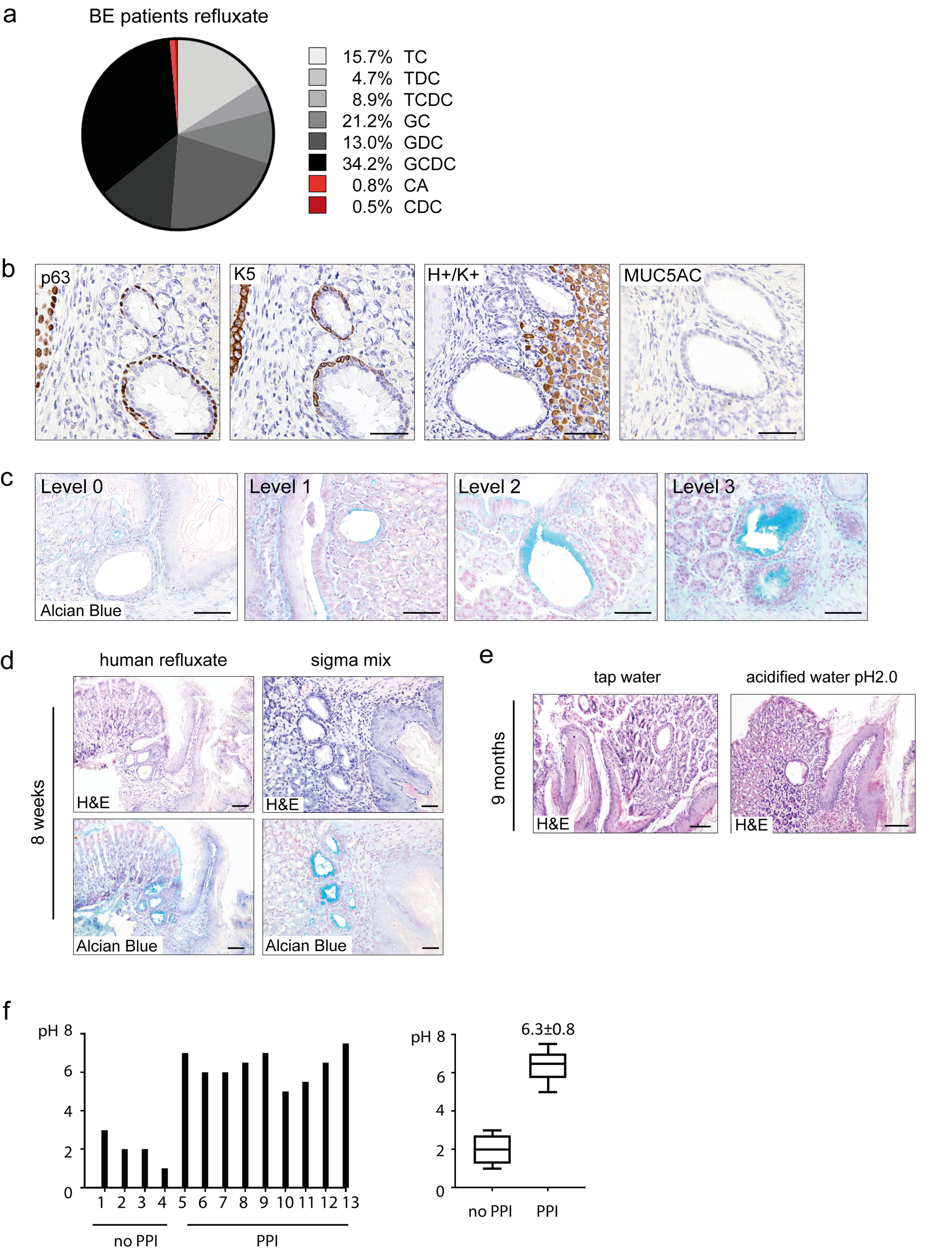

Supplement: S2 Fig — [A] Percentage of each individual bile component found in BE patients refluxates as analyzed by high performance liquid chromatography (HPLC) “[51]”. [B] IHC for squamous markers p63 and K5 and columnar markers H+K+ (parietal cells) and MUC5AC (gastric pit cells) in multilayered glandular structures (MLGS) at the SCJ in mice after treatment with BE patients refluxates for 8 weeks (n = 50). [C] Alcian blue expression level, representing the secretion of acidic mucins, in the MLGS at the SCJ in mice after bile treatment. 0 = Alcian blue absent, no secretion of acidic mucins; 3 = high levels of Alcian blue staining inside the MLGS representing massive acidic mucin secretion. [D] The 6 individual conjugated BAs were purchased from Sigma and mixed together in the same ratio (TC:TDC:TCDC:GC:GDC:GCDC, 3:1:2:4:3:7) as found in human refluxates. Oral gavage of the sigma mix (pH ~7) was as effective as the human refluxate samples in inducing gland development at the SCJ in (6/6) mice, in both number of glandular structures and mucin production. [E] Development of MLGS at the SCJ in mice after 9 months on normal tap water and acidified drinking water pH2.0. [F] pH of human refluxates with (6.3±0.8) or without (2.0±0.8) taking PPIs. Scale bars 100μm. (TIF) [file pone.0220050.s002.tif]

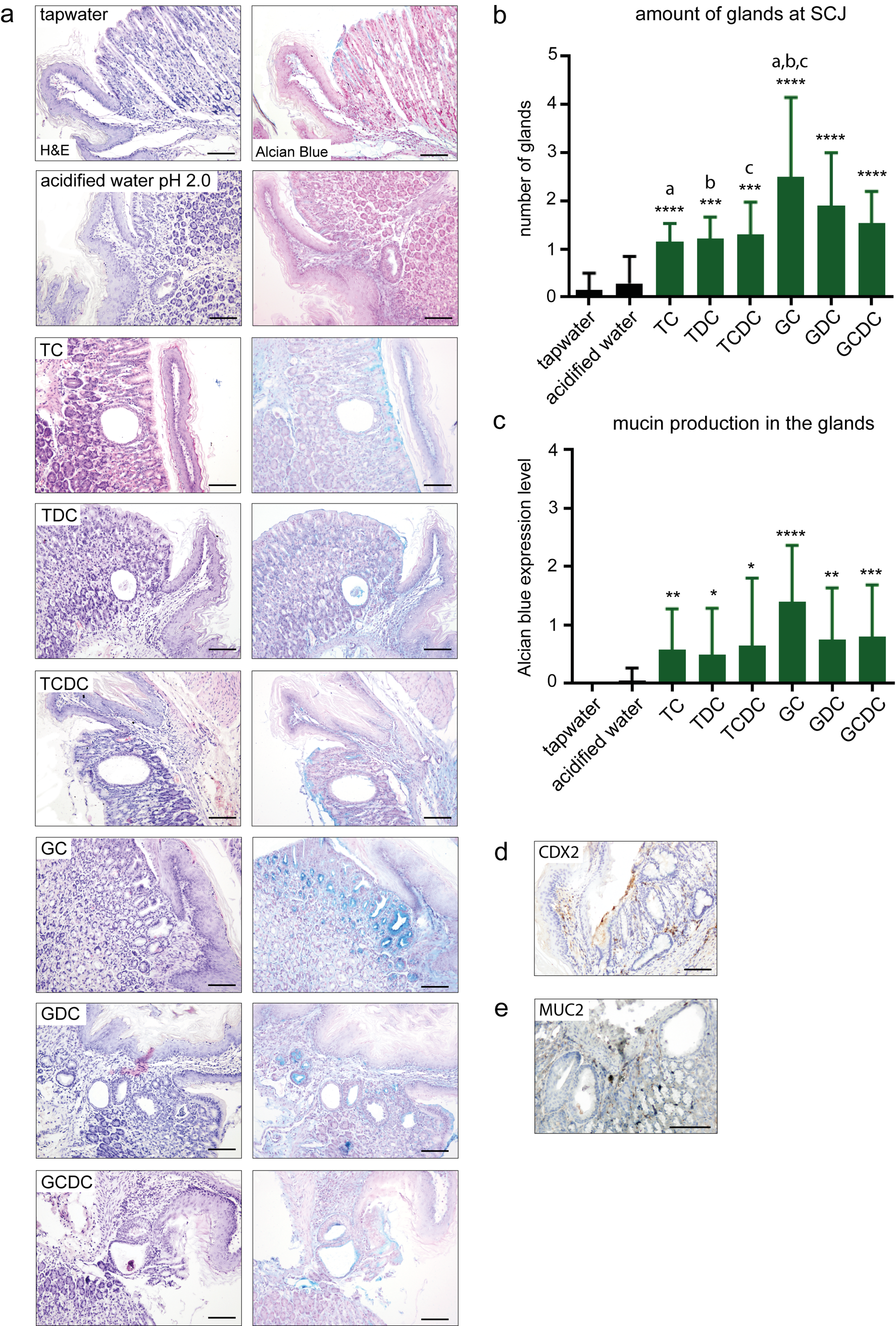

Supplement: S3 Fig — [A] H&E and Alcian blue staining of the SCJ in mice treated with individual bile components (10mM) for 8 weeks resulting in the development of MLGS (n = 6 per bile component). [B] Quantification of number of glandular structures and [C] the secretion of acidic mucins, represented by the amount of Alcian blue staining inside the glandular structures (S2C Fig), at the SCJ in mice treated with individual BAs for 8 weeks. Treatment with individual bile components was compared to treatment with acidified water pH 2.0. Data are represented as mean±SD. Unpaired t test *p<0.05, *p<0.01, ***p<0.001. Individual components GC showed significant more glandular structures at their SCJ compared to TC, TDC and TCDC (a, p = 0.009; b, p = 0.038; c, p = 0.047). No differences were seen in Alcian Blue expression inside the glands. Taurocholic (TC), Taurodeoxycholic (TDC), Taurochenodeoxycholic (TCDC), Glycocholic (GC), Glyco-deoxycholic (GDC) and Glycochenodeoxycholic acid (GCDC). [D] IHC for intestinal marker CDX2 in MLGS at the SCJ in mice. [E] IHC for intestinal marker MUC2 in MLGS at the SCJ in mice treated with taurine conjugated bile acid. Scale bars 100μm. (TIF) [file pone.0220050.s003.tif]

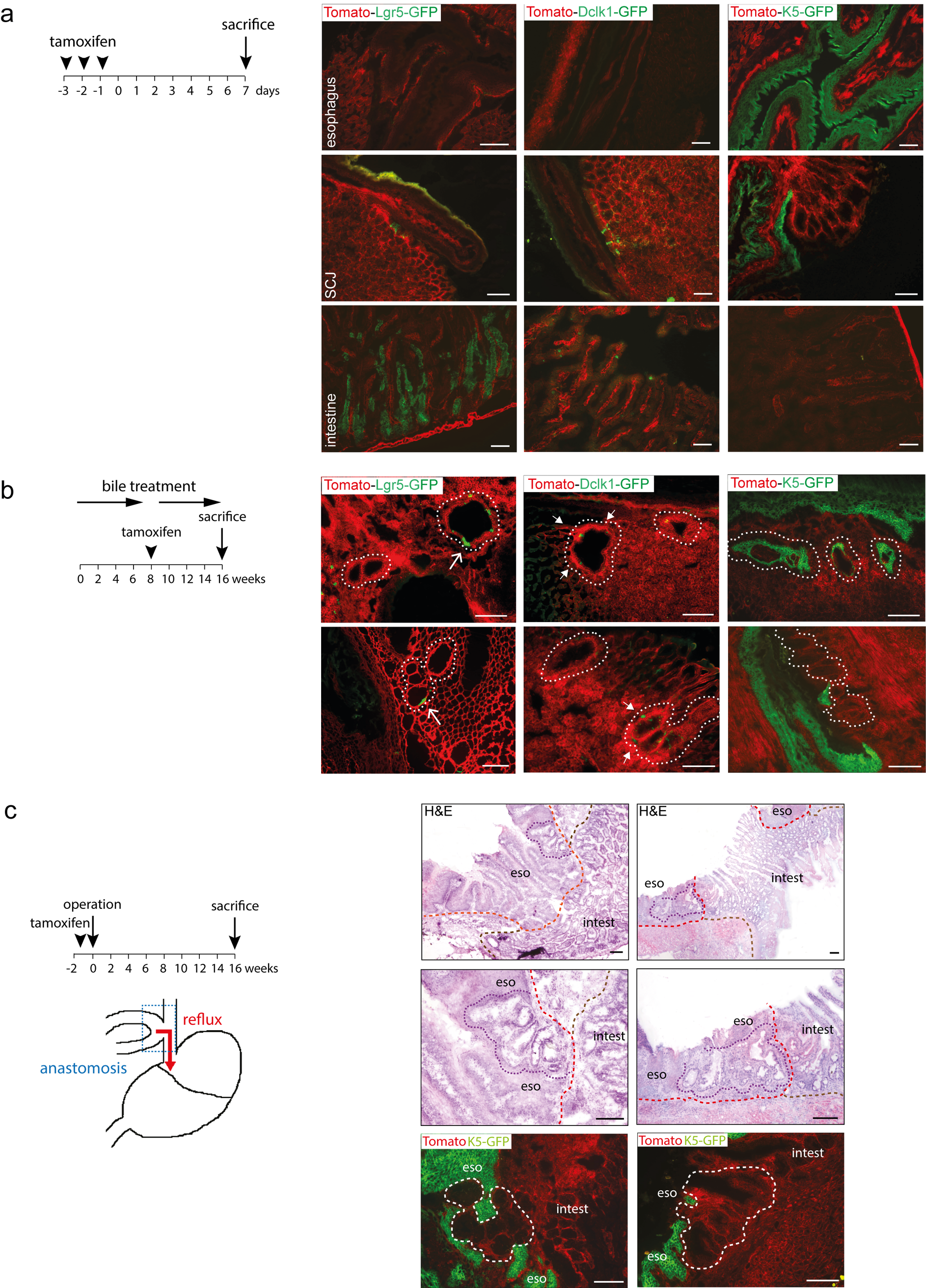

Supplement: S4 Fig — [A] Expression of Lgr5-GFP, Dclk1-GFP and K5-GFP positive cells in the normal esophagus, at the normal SCJ and in the intestine: Lgr5-cre (n = 5) “[29]”, Dclk1-cre (n = 5) “[27]” and K5-cre (n = 5) “[52]” mice crossed with Rosa26-Tomato-GFP mice were injected with Tamoxifen for 3 days and sacrificed after 7 days to see the expression in normal esophageal, SCJ and intestinal tissue. [B] Expression of K5-GFP, Dclk1-GFP (white arrows) and Lgr5-GFP (white double arrows) positive cells in MLGS at the SCJ in mice (white dotted areas) after: Lgr5-cre-Tomato-GFP (n = 10), Dclk1-cre-Tomato-GFP (n = 10) and K5-cre-Tomato-GFP (n = 10), mice were treated with BE patients refluxate for 8 weeks to allow development and multiplication of the MLGS at the SCJ. After 8 weeks, mice were injected with tamoxifen for 3 days and treated with bile acids for another 8 weeks. [C] Tamoxifen injection (3 days, 0,25mg i.p.) followed by surgically induced reflux resulted in metaplastic area (purple or white dotted line) at the anastomotic site which was negative for K5-GFP positive cells. The squamous tissue, eso (esophagus, red dotted area) was positive for K5-GFP positive cells. Scale bars 100μm. (TIF) [file pone.0220050.s004.tif]
